# Inadequate positioning of central venous catheters inserted at intensive care units

**DOI:** 10.31744/einstein_journal/2022AO6497

**Published:** 2022-03-29

**Authors:** Álisson Vinicius dos Santos, Edson Dias Barbosa, Geraldo Vicente Nunes, Raquel da Silva Cavalcante, Ayanne Karla Ferreira Diniz, Gustavo Rocha Costa Freitas, Jaqueline Figueirôa Santos Barbosa de Araújo

**Affiliations:** 1 Real Hospital Português de Beneficência em Pernambuco Recife PE Brazil Real Hospital Português de Beneficência em Pernambuco, Recife, PE, Brazil.

**Keywords:** Catheters, Radiography, thoracic, Central venous catheters, Patient positioning

## Abstract

**Objective:**

To evaluate the positioning of the distal tip of central venous catheters and the factors that contributed to inadequate positioning in patients admitted to intensive care.

**Methods:**

This is a cross-sectional study, with a sample of 246 medical records of patients admitted to intensive care units. A catheter position analysis form was used as an instrument for data collection.

**Results:**

It was seen that 86.2% of catheters used in intensive care were centrally inserted in the internal jugular veins, 74.4% were double-lumen catheter, and ultrasound was employed for puncture technique in 84.6% of cases. Of the distal ends of the catheters, 53.7% were at the cavoatrial junction (correct position). According to statistical tests, there was a positive correlation between the inadequate positioning of the distal extremity with the central insertion catheter (p=0.012). Patients with presumptive diagnosis associated with COVID-19 showed a positive correlation with inappropriate positioning of the catheter distal tip (p=0.017).

**Conclusion:**

There are extrinsic factors related to improper positioning of the distal tip of catheters, such as the type of catheter used, the patients’ diagnosis and the puncture with insertion in the left jugular vein.

## INTRODUCTION

Critically ill patients admitted to intensive care units (ICU) and emergency departments are submitted to central insertion venous catheters (CICC), which is considered the most common invasive procedure performed in critically ill patients.^(1,2)^ The use of CICC has been growing as a result of the increasing number of critically ill patients who require safer, long-term vascular access.^(1)^

Central insertion venous catheters are devices made of radiopaque polyurethane with a tapered distal tip, and can be divided into single-, double-, or triple-lumen. The lumens are further identified as proximal, medium, and distal, depending on their quantity. Central insertion venous catheters are inserted by puncturing a central vein (internal jugular, subclavian, axillary or femoral), or by inserting into peripheral vein (cephalic, basilic, or brachial), being considered as central venous access when the tip is positioned close to the cavoatrial junction (CAJ).^(3)^

Central insertion venous catheters are fundamental parts of vascular access, and are necessary for administration of solutions with pH<5.0 or >9.0; osmolarity >500mOsm/L or vesicant characteristics; parenteral nutrition; rapid replacement of liquids or blood; hemodynamic monitoring and renal replacement therapy.^(1,4,5)^

However, use of CICC is associated with several complications, the frequency of which reaches 20%.^(4)^ Among these complications, infectious and thrombotic processes, pneumothorax, and inadequate positioning of the catheter distal tip stand out.^(6)^

Central insertion venous catheters with poor positioning of the distal tip may increase the risk of complications for the patient, such as pneumothorax; thrombosis, and arrhythmias; infusion of solutions incompatible with the vessel; extravasation of vesicant drugs; tricuspid valve damage, and cardiac tamponade due to auricle perforation.^(6,7)^ The rate of poor positioning of the CICC varies from 6.8% to 87.7%.^(2,6)^ The discussion about the ideal method of tip location is intense, but it should be located in an area below the carina, up to near the middle of the right atrium, where the superior vena cava has its greatest diameter and, consequently, the greatest blood flow. In this area, irritating or vesicant medications do not have contact with the endothelium of the vein long enough to cause damage.^(2,6,7)^

Traditionally, the most used technique to perform the puncture of central thoracic veins is based on anatomical references. For puncture of the jugular veins, the reference is the apex of the meeting of the branches of the sternocleidomastoid muscle and the lateral surface of the carotid artery. For the puncture of the subclavian veins, the anatomical landmark is 1cm below the transition point of the medial third of the clavicle with the mid third.^(2)^

During insertion of the catheter without resources, such as ultrasound or electrical navigation devices, physician use literature recommendations on the appropriate length of catheter insertion per site, but these measurements are completely empirical considering the great variability of biotypes (longilineal, brevilineal, obese, thin, etc.). To overcome this difficulty, some centers recommend inserting a length longer than needed, and, after checking the tip on chest radiography, evaluating whether it should be pulled or not.^(2,7)^

There are several published studies, including meta-analyses, proving the use of real-time ultrasound for CICC insertion is the safest technique, because it ensures visualization of the needle and vein throughout the puncture. However, the availability of portable ultrasound equipment in hospitals is still low.^(2)^

The gold standard method for detecting inadequate CICC positioning is by means of post-insertion chest radiography.^(2,7)^ For the CICC to be considered in adequate position, it must be located in the CAJ area, which corresponds to the transition area between the right lateral margin of the superior vena cava (usually a vertical line) and the right lateral margin of the right atrium (usually a curved line).^(6-8)^

## OBJECTIVE

To evaluate the position of the distal tip of central venous catheters and the factors that contributed to inappropriate positioning in intensive care patients.

## METHODS

### Study population

This is a cross-sectional epidemiological study developed at a hospital in the city of Recife (PE). The study sample was composed of medical records of patients submitted to CICC puncture in general and specialized (neurological, cardiological, nephrological, and cardiothoracic recovery unit) ICUs, from March to May 2020.

The study sample comprised medical records of patients of both sexes, aged over 18 years, who had received some type of CICC. Patients’ charts with incomplete information and without the first chest radiograph after CICC insertion were excluded.

### Data collection

The data were collected from the electronic medical record system and from the report of the catheter committee of a tertiary referral hospital, in the city of Recife, which contains data of discharged patients. To retrieve information regarding the positioning of the distal tip of the CICC, the first chest radiograph with an anteroposterior view was used, right after the insertion of the central catheter.

The literature and the organizational protocol for adequate positioning of the distal tip of the CICC were taken as a basis, which consider as appropriate location all catheters that are in the CAJ area.^(6,8)^

The order of collection was based on an Excel spreadsheet made available by the catheter commission team, which had the names and records of the patients. The collection followed the order of the first medical record and then followed the sequence; if any selected medical record did not meet the inclusion criteria of the study, the subsequent medical record would be selected.

### Instruments

The information from each medical record, as well as the data regarding patients and CICC were collected using an instrument developed by the author and made available through the Google Drive form.

To prepare the data collection instrument, a bibliographic search was carried out. And together with the Catheter Committee, composed of physician and nurse of the infusion therapy service, a discussion was held about the main variables that influence the positioning of the CICC. To validate the instrument, a pilot study was conducted with 10% of the sample, that is, 24 medical records, with the purpose of verifying the difficulties. The collection instrument consisted of a self-explanatory form, composed of multiple-choice questions, with two subdivisions for collection, making it possible to recognize the profile of the selected sample, as well as that of the CICC.

For the sample profile, the following factors were observed: age; sex (male and female); body mass index (BMI), with data collected from the nutritional assessment form, and presumptive diagnosis, grouped by specialty clinic. Regarding the catheter, the selected type (CICC or peripherally inserted central catheter - PICC), the number of lumens, insertion site, position of the catheter’s distal tip, number of attempts, and the puncture technique used.

### Data analysis

The data collected were duly recorded in a pre-coded database for input in Excel 2016 software, and then analyzed using the (SPSS, IBM, Armonk, New York, United States), version 21.0. For the analysis, the relative and absolute frequencies of the classes of each qualitative variable were calculated. For the quantitative variables, means and medians were used to summarize the information, in addition to standard deviations, minimum and maximum values, to indicate data variability. The significance level set was 5%. The statistical analyses were performed using SPSS software, version 21.0.

### Ethical aspect

This study was approved by the Research Ethics Committee of the *Real Hospital Português de Beneficência em Pernambuco* (RHF), with CAAE: 29771920.4.0000.9030 and opinion #4.070.627. The Informed Consent Form was not necessary, considering the research was documental, descriptive, and retrospective.

## RESULT

The study had a sample of 246 patient records (61% were male). The age of the participants ranged from 18 to 99 years, with a mean of 66.4 years and a standard deviation of 16.7. Regarding the sample profile, the patients admitted to ICU had a mean BMI of 27.7*, i.e*., classified as overweight, ranging from 16.3 to 46.8.

According to the statistical tests, there was no positive correlation between inadequate positioning of the distal tip of the CICC and the variables sex (p=0.289), age (p=0.595), and BMI (0.439).

Table 1 shows the distribution of variables and results according to catheter type, number of lumens, insertion site, position of the catheter distal tip, number of attempts, and puncture technique used.

**Table 1:** Evaluation of information regarding central venous catheters

Variables	n (%)
Type of catheter	
CICC	212 (86.2)
PICC	34 (13.8)
Number of lumens	
Single	6 (2.4)
Double	183 (74.4)
Triple	57 (23.2)
Puncture technique	
Anatomical landmarks	38 (15.4)
Guided by ultrasonography	208 (84.6)
Number of puncture attempts	
One	106 (43.1)
Two or more	140 (56.9)
Insertion site	
RIJV	98 (39.8)
LIJV	65 (26.4)
Subclavian veins	50 (20.3)
Veins of the arm	33 (13.5)
Position of the distal extremity (tip of the catheter)	
Adequate (cavoatrial junction)	132 (53.7)
Inadequate	114 (46.3)
Total	246 (100)

CICC: centrally inserted central catheter; PICC: peripherally inserted central catheter. RIJV: right internal jugular vein. LIJV: left internal jugular vein.

Table 2 shows there was a statistically significant association between the distal tip of the catheter position and the type of catheter used (p=0.012). Patients with CICC had a higher percentage of inadequate positioning of the distal tip when compared to those with PICC. At this hospital, most of the PICC insertions were performed by nurses, who used a more objective measurement technique to assess the length of the catheter to be introduced, while physicians made an empirical assessment. There was a statistically significant association between catheter position and insertion site (p<0.05). The left internal jugular vein (LIJV) showed the highest percentage of catheter inadequacy. The greater distance from the insertion site of the LIJV to the CAJ, which is on the right side of the chest, justifies this finding. In this same analysis, 72.7% of insertions made in the arm were well positioned; this is the PICC insertion site used most by nurses.

**Table 2:** Evaluation of the association between catheter and insertion site data and distal tip positioning

Variables	Positioning of the distal tip of the catheter			p value^*^

	Inadequate n (%)	Adequate n (%)	Total n (%)	
Type of catheter				0.012
CICC	105 (49.5)	107 (50.5)	212 (100.0)	
PICC	9 (26.5)	25 (73.5)	34 (100.0)	
Puncture technique				
Anatomical landmarks	22 (57.9)	16 (42.1)	38 (100.0)	0.120
USG	92 (44.2)	116 (55.8)	208 (100.0)	
Number of punctures				
One	49 (46.2)	57 (53.8)	106 (100.0)	0.837
Two or more	65 (53.8)	75 (46.2)	140 (100.0)	
Insertion site				
RIJV	39 (39.8)	59 (60.2)	98 (100.0)	0.001
LIJV	46 (70.8)	19 (29.2)	65 (100.0)	
Subclavian veins	20 (40.0)	30 (60.0)	50 (100.0)	
Veins in the arm	9 (27.3)	24 (72.7)	33 (100.0)	

* By means of Pearson’s χ^2^ test.

CICC: centrally inserted central catheter; PICC: peripherally inserted central catheter; USG: ultrasonography; RIJV: right internal jugular vein; LIJV: left internal jugular vein.

There was a positive correlation between a higher percentage of poor catheter positioning and the presumptive diagnosis of lung disease related to coronavirus disease 2019 (COVID-19), p=0.017.

## DISCUSSION

The study sample was composed mostly of male patients with a mean age of 66.4 years and a BMI of 27.7 (overweight). Some studies^(1,9,10)^ published the mean age of patients requiring a central catheter is above 60 years. This is justified by the fact that, with advancing age, there is also an increase in chronic diseases, as well as their complications, which may lead to ICU admission. Regarding BMI, a retrospective single center study showed patients with a low BMI had fewer catheter-related mechanical complications, such as inadequate positioning.^(10)^

The prevalence of inappropriate CICC positioning ranges from 6.8% to 87.7%, *i.e*., it is a wide range due to several definitions of correct positioning.^(2,6)^The sample of the present study found 46.3% of poor positioning, according to the organizational protocol. Patients who underwent CICC puncture and presented as mechanical complication an inadequate positioning of its distal tip, may manifest some clinical signs suggestive of such a complication, such as chest pain associated with fluid infusion through CICC, retrosternal pain, headache and “bubbling” in the ears or head.^(11)^

In the present study, there was an increase in the number of CICC (212; 86.1%), as well as a positive association regarding the inadequate positioning of the CICC tip. The large number of CICC with inadequate positioning is related to the fact that most patients in the ICU have greater clinical instability, and CICC is the most indicated because it is an emergency access. One study demonstrated that, despite the skill level of the professionals and the technique used, the inadequate positioning of the CICC is linked to the influence of congenital and acquired anatomical variants.^(11)^Peripherally inserted central catheter, however, has a poor positioning rate of 20%,^(12,13)^and this puncture is more indicated when the patient has greater clinical stability.

The main CICC puncture sites were the internal jugular veins (165; 66.5), but the LIJV was the one that presented a positive correlation of inadequate positioning (p<0.05). Studies showed jugular veins are the main veins to be chosen for CICC puncture.^(1,4,9)^The insertion site is chosen according to the professional’s assessment and degree of skill, and their preference for jugular veins may be highlighted due to easy access and lower risk of eventual complications. However, incorrect CICC positioning is more common in the LIJV, due to anatomical variations of left thorax veins.^(11)^

The main puncture technique used during the insertion of CICC and PICC was ultrasonography (208; 84.5%). The present study presents a percentage of ultrasonography use for punctures higher than the average found in literature.^(1)^ This is because the study site is a private hospital, in which the technological resources of ultrasonography are more readily available. The Catheter Committee of the hospital developed an organizational protocol, a training program for physicians in echo-guided puncture, thus performing a surveillance of the punctures. The protocol recommends all PICC insertions be performed by ultrasonography, and elective CICC insertions be performed by ultrasonography at the internal jugular vein and femoral vein sites, accepting the anatomical landmarks for the subclavian veins, due to the greater technical difficulty of echo-guided puncture at this site. Table 1 shows 86.4% of CICC and PICC insertions were done by ultrasound. A systematic literature review study conducted in 2018 showed there was an increase in the assertiveness rate of punctures guided by ultrasound compared to those guided by anatomical landmarks.^(14)^ This increase in assertiveness rate is linked to the association of technological resources for better visualization of blood vessels during puncture, providing greater safety to the professional and the patient. Cases of great technical limitation or need for sedation (for patients in wards) are performed in the operating room guided by fluoroscopy.

The hospital’s Catheter Committee established good practices for preventing complications. Thus, 74.4% were double-lumen, with only 23.2% triple-lumen – the latter is associated with the highest risk of bloodstream infection.

The main diagnoses found in the ICU environment were related to pulmonology, infectious diseases, nephrology, and COVID-19. There is a significant correlation of inadequate catheter positioning with diagnosis of COVID-19 (p=0.017). The literature shows that certain complications, such as pulmonary collapse or pleural effusions, may deviate venous structures, such as the distal tip of the CICC, from their proper location.^(11)^ In early March 2020, the World Health Organization (WHO) declared a pandemic state of COVID-19, with an exponential increase in the number of ICU admissions due to complications of the disease. The population aged over 60 years and with prior comorbidities was more susceptible to a worse prognosis for COVID-19.^(15)^

With the emergence of COVID-19, there were also several doubts about management of these patients, requiring the development of protocols for rapid sequence of orotracheal intubation and invasive procedures, to reduce the risk of contamination and spread of the virus among healthcare professionals. Due to the higher complexity and clinical instability of COVID-19 patients, the selection of a vascular access was preferably done by using a CICC with more than one lumen, for fluid support, vasopressor administration, parenteral nutrition, and hemodynamic monitoring.^(16)^

Peripherally inserted central catheter show superior performance when compared to CICC, since they have advantages, such as insertion site in the arm region (a site further from the patient’s mouth and nose, thus reducing the risk of contamination) and more appropriate handling for respiratory care (noninvasive ventilation, tracheostomies, or periodic pronation).^(16)^ It is worth noting that the choice of CICC depends on the healthcare professionals and the patient’s clinical condition.

As limitations of this study, we point out the level of expertise of the healthcare professional that punctured the catheter, the height of the patients, and the measurement introduced of the catheter were not investigated. It is suggested that new studies be carried out to demonstrate possible correlations between inadequate positioning and these variables.

## CONCLUSION

Some factors may contribute to inadequate positioning of the distal tip of central venous catheters, such as the type of catheter used, presumptive diagnoses, and puncture of the left internal jugular veins. The appropriate location of the catheter tip represents an important technical aspect in the modern, evidence-based era. The insertion technique used cannot be ignored when assessing the incidence and impact of central venous catheter complications. Understanding the mechanisms that lead to inadequate positioning of central venous catheters, as well as its prevention, identification, and correction, is of utmost importance to reduce health care-related harm to patients admitted to intensive care units or emergency rooms.
